# Isolation and Identification of a Rumen *Lactobacillus* Bacteria and Its Degradation Potential of Gossypol in Cottonseed Meal during Solid-State Fermentation

**DOI:** 10.3390/microorganisms9112200

**Published:** 2021-10-21

**Authors:** Wei-Kang Wang, Wen-Juan Li, Qi-Chao Wu, Yan-Lu Wang, Sheng-Li Li, Hong-Jian Yang

**Affiliations:** State Key Laboratory of Animal Nutrition, College of Animal Science and Technology, China Agricultural University, Beijing 100193, China; 18292092306@163.com (W.-K.W.); liwjuan1226@163.com (W.-J.L.); wuqichao@cau.edu.cn (Q.-C.W.); wang_yanlu@cau.edu.cn (Y.-L.W.); lisheng0677@163.com (S.-L.L.)

**Keywords:** gossypol, rumen, solid-state fermentation, cottonseed meal

## Abstract

Cottonseed meal (CSM) is an important protein feed source for dairy cows. Its inclusion in ruminant diets is limited due to the presence of the highly toxic gossypol though rumen microorganisms are believed to be capable of gossypol degrading and transforming. The objective of the present study was to isolate the gossypol-degrading bacteria from the rumen contents and to assess its potential for gossypol degradation in vitro. A strain named *Lactobacillus agilis* WWK129 was anaerobically isolated from dairy cows after mixed rumen microorganisms were grown on a substrate with gossypol as the sole carbon source. Furthermore, the strain was applied at 5% inoculum concentration in vitro to continuously ferment CSM at 39 °C for five days, and it presented gossypol degradability as high as 83%. Meanwhile, the CSM contents of crude protein, essential amino acids increased significantly along with the increase of lactic acid yield (*p* < 0.01). Compared with the original CSM, the fermented CSM contents of neutral detergent fiber and acid detergent fiber was remarkably decreased after the anaerobic fermentation (*p* < 0.01). In brief, the *Lactobacillus* strain isolated from the rumen is not only of great importance for gossypol biodegradation of CSM, but it could also be used to further explore the role of rumen microorganisms in gossypol degradation by the ruminants.

## 1. Introduction

**Figure 1:**
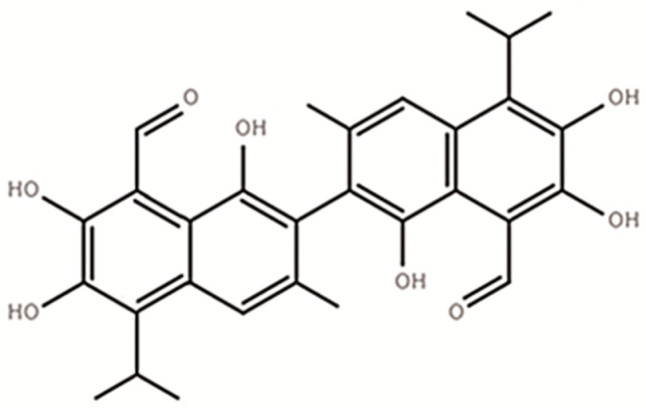
The structure of free gossypol (C_30_H_30_O_8_).

Gossypol (C_30_H_30_O_8_), a polyphenolic compound (Figure 1) produced by the cotton plant (*Gossypium* sp.), is one of the anti-nutritional factors to limit the application of cotton by-products in animal production [1,2]. For a long time, excessive intake of gossypol has been found to cause growth depression [3], reproductive performances decline, anemia, as well as other internal organ abnormalities in animals [4,5]. Adult ruminants were believed more tolerant to gossypol than monogastric animals as well as young ruminants [6]. Such tolerance was believed to be associated with the characteristics of gossypol binding to soluble proteins and gossypol partially degrading ability of rumen microbes [7,8].

As for cottonseed meal (CSM), an important protein feedstuff, three methods have been commonly used in animal feed processing for many years to reduce gossypol’s toxicity, including i) mechanical processing [9], ii) chemical treatment [10,11], and iii) microbial fermentation [12]. Among these methods, microbial fermentation was considered the most promising method for gossypol detoxification as well as a nutritional improvement compared with the other methods with subsiding effects, especially for CSM [13,14]. Numerous fungi were reported to play an important role in CSM fermentation for gossypol degradation, including *Candida tropicalis*, *Saccharomyces cerevisiae*, *Aspergillus oryzae*, *Aspergillus terreus*, and *Aspergillus niger* [15,16]. Although these fungi were found capable of gossypol degradation, some fungi metabolites were toxic to animals, e.g., citrinin secreted by *Aspergillus* species [17]. Thus the safety of fermentation products by fungi generally used as animal feeds must be assessed. To overcome the above constraints of extrinsic environmental microorganisms, the authors in the present study hypothesized that there should be some endogenous microbes capable of gossypol degradation in the rumen. Therefore, the objectives of the present study attempted to isolate potential microorganism strain with gossypol degradation ability from rumen contents, and CSM was chosen as a mode gossypol containing feed to investigate if the strain could rebuild the nutritional nature of CSM.

## 2. Materials and Methods

### 2.1. Isolation of Gossypol Degradation Bacteria

**Figure 2:**
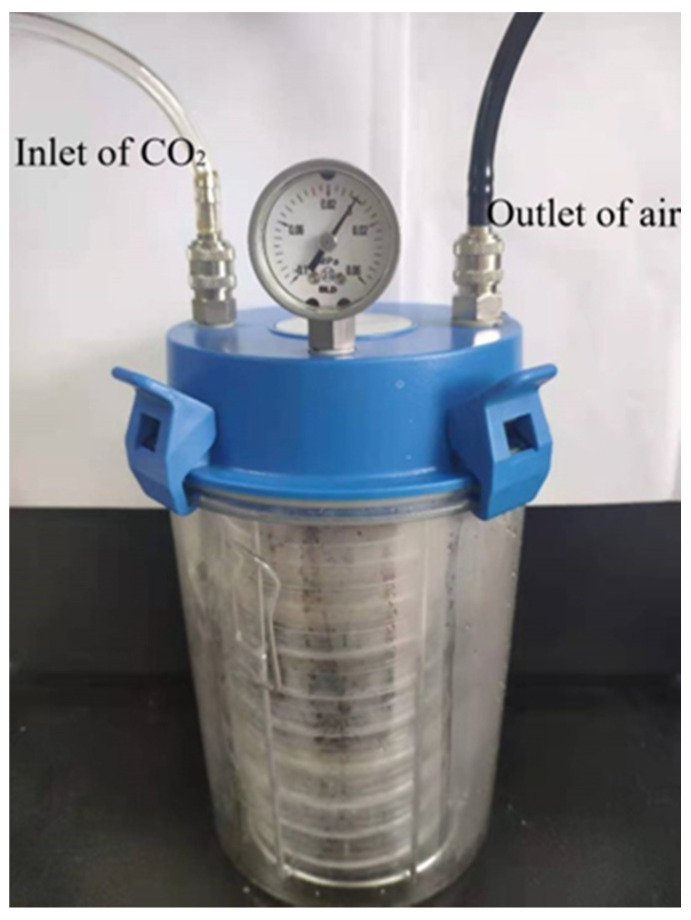
Instruction of anaerobic jar.

Rumen liquid was collected from the rumen central of three mature lactating Holstein cows with ruminal cannulas (average body weight: 502 ± 25 kg, 151 days in milk and 36 kg/d of milk yield at the beginning of the study), and filtered through four layers of cheesecloth. Cows were individually housed in three stalls with good ventilation and were fed 25 kg of dry matter (DM) (110 g/kg of imported Alfalfa, 490 g/kg of silage maize, 140 g/kg of pressed corn, and 260 g/kg of mixture) per animal per day, and free access to drinking water. The cows were fed at 09:00 am and 15:00 pm, and milked three times daily. The filtrated rumen fluids from the three animals were mixed in equal proportion. Approximately 1 mL of the rumen liquid was diluted with saline solution to make 10^−1^ and 10^−2^ dilutions of rumen liquid, 1 mL of different concentrations of diluted rumen liquid were evenly inoculated onto basal medium plates (5 g/L (NH_4_)_2_SO_4_, 1 g/L KH_2_PO_4_, 1 g/L NaCl, 0.5 g/L MgSO_4_.7H_2_O, 0.1 g/L CaCl_2_, 0.2 g/L yeast extract, 15 g/L agar) [16] containing 1 g/L gossypol as the sole carbon source and then cultured at 39 °C for three days using AY6907 anaerobic jar (GeneScience, Wilmington, USA) which reached anaerobic condition by the inlet of CO_2_ to remove O_2_ (Figure 2). The isolated strain was identified and inoculated on DeMan-Rogosa-Sharpe (MRS) medium (10 g/L peptone, 20 g/L glucose, 5 g/L yeast extract, 4 g/L K_2_HPO_4_, 5 g/L CH_3_COONa, 10 g/L beef extract, 2 g/L C_6_H_5_O_7_(NH_4_)_3_, 1 mL/L Tween-80, 0.56 g/L MgSO_4_.7H_2_O, 0.14 g/L MnSO_4_.H_2_O, 15 g/L agar) [18] and incubated for three days at 39 °C to obtain pure cultures. The isolated strain was maintained at 4 °C on MRS medium.

### 2.2. Morphological Characteristics of the Strain

The isolated strain was cultured in a liquid MRS medium for 24 h at 39 °C under anaerobic condition. Then the thallus was harvested and prefixed with a 2.5% glutaraldehyde solution overnight at 4 °C. After it was prefixed, the thallus was washed with 0.1 M sodium phosphate buffer solution (pH 7.0) three times and serially dehydrated with 50%, 70%, 80%, 90%, and 100% ethanol, respectively. Then, thallus was dried at the critical point. For scanning electron microscope (SEM), a thin film of thallus was sputter-coated with a thick gold film and observed with a HITACHI UHR FE-SEM SU8020 (Hitachi, Tokyo, Japan) SEM. For transmission electron microscope (TEM), thallus was treated with osmium tetroxide solution for 30 min, and washed in phosphate buffer, dehydrated in ethanol gradients as described above, embedded in Epon-Araldite resin for making the blocks of the cells pellet. Ultra-thin sections of the thallus were stained in uranyl acetate and lead citrated, air-dried, and observed with a JEOL JEM-1200EX (Jeol, Tokyo, Japan) TEM [19].

### 2.3. DNA Extraction and Bacteria Identification

After the harvest of thallus as described in Section 2.2, the extraction of DNA was carried out by using the QIAamp^®^ DNA stool mini kit (Qiagen Ltd., Crawley, West Sussex, UK) following the manufacturer’s instructions. The concentration of total DNA was estimated using a Nanodrop 2000 (Thermo Scientific, Wilmington, USA) and purified DNA samples were stored at –80 °C. The genomic DNA was amplified by polymerase chain reaction (PCR) for use as a template. The primers were 27F: 5′-AGAGTTTGATCCTGGCTCAG-3′ and 1492R: 5′-ACGGTTACCTTGTTACGACTT-3′ [20], and the PCR reaction was conducted using the following conditions: 2 min at 98 °C, followed by 35 cycles of 10 s at 98 °C, 10 s at 56 °C, and 15 s at 72 °C, and a final extension of 5 min at 72 °C.

The isolated strain 16S rDNA gene was sequenced by Beijing TSINGKE, China. The product of 16S rDNA genes of the isolated strain in the present study was compared against National Center for Biotechnology Information database (NCBI) reported sequences with the Basic Local Alignment Search Tool (BLAST) hosted at the website of NCBI. A phylogenetic tree was then drawn using the Neighbor-joining method [21]. Phylogenetic and molecular evolutionary analysis were conducted at 1000 bootstrap value using Molecular Evolutionary Genetics Analysis (MEGA) version 5.0 software (Center of Evolutionary Functional Genomics, Biodesign Institute, Arizona State University, Tempe, AZ, USA) [22].

### 2.4. Growth Study of Isolated Bacteria in Different Carbon Source

Growth experiments were conducted with different carbon sources. After the isolated strain was anaerobically cultured in MRS medium for 24 h as described in Section 2.2, 1 mL cultures were inoculated onto 500 mL liquid MRS medium which used glucose or gossypol (1 g/L) as carbon resource, respectively. Then, the inoculated MRS mediums were mixed well and divided 2 mL into each tube. All of these tubes were anaerobically incubated at 39 °C, bacteria growth of 3 tubes was determined by optical density at 600 nm every 1 h using a spectrophotometer.

### 2.5. Solid-State Fermentation and Chemical Analysis

A representative CSM sample that contained 500 mg/g gossypol was dried at 65 °C for 24 h in a forced-air oven and ground to pass through a 0.25-mm screen. The nutritive composition of CSM sample included 164 g/kg of neutral detergent fiber (NDF), 115 g/kg of acid detergent fiber (ADF) and 500 g/kg of crude protein (CP) in DM. Then the CSM sample was autoclave sterilized at 121 °C for 25 min. The isolated strain was cultured in liquid MRS medium for 24 h at 39 °C under anaerobic condition, and 1.5 mL cultured medium was diluted to 15 mL by liquid MRS medium. For each fermentation period, a 15 g CSM sample was inoculated with 15 mL of the diluted cultured medium in each 10 cm culture dish, resulting in 5% inoculum level and 50% moisture in dry matter (DM). All of these culture dishes (5 incubation time × 3 replicates) were put in a anaerobic jar, purged with CO_2_ to obtain anaerobic condition, and anaerobically incubated at 39 °C for 1, 2, 3, 4, and 5 days, respectively. Meanwhile, to prevent the confusion of the effect of medium contents on fermentation results, a 15 g CSM sample was inoculated with 15 mL liquid MRS medium without isolated strain in three replicates and incubated at the same conditions as above for five days.

After incubation was completed, CSM samples were dried in an oven at 65 °C for 48 h for later analysis. Crude proteinwas determined according to AOAC methods [23], amino acids were assayed using a Hitachi L-8800 instrument (Hitachi, Tokyo, Japan). Neutral detergent fiber and acid detergent fiber were determined following the method of Van Soest et al. [24]. Lactic acid was determined by High Performance Liquid Chromatography (HPLC) according to the methods of Cira et al. [25]. Gossypol content was determined as the methods of Wang et al. [26]: 1.5 g sample in 15 mL acetone was ultrasound 30 min at 40 °C, centrifuged and collected the supernatant, repeat the above processes three times, the extraction was combined and filtrated with 0.45 μm microporous, rotary evaporated, dissolved by acetonitrile −0.2% phosphoric acid solution and fixed capacity to 2.5 mL. The content of gossypol was quantified by HPLC with a Wufeng analytical instrument (Wufeng Co., Ltd., Shanghai, China). The analytical column was a symmetry reversed-phase C18 column (250 × 4.6 mm, 5 μm, pH 2–8, Waters, Milford, MA, USA). The mobile phase was 85:15 (*v*/*v*) acetonitrile −0.2% phosphoric acid solution at a flow rate of 1 mL/min. Injections were 20 μL, and the gossypol was detected at 235 nm.

### 2.6. Statistical Analysis

Statistical analyses were completed using the general linear model procedure of SAS [27]. Standard errors (S.E.M) of means, adjusted by the Tukey method, were estimated by the least square means procedure of SAS [27]. Significance was declared at *p* < 0.05 unless otherwise noted.

## 3. Results

### 3.1. Morphological Characteristics

**Figure 3:**
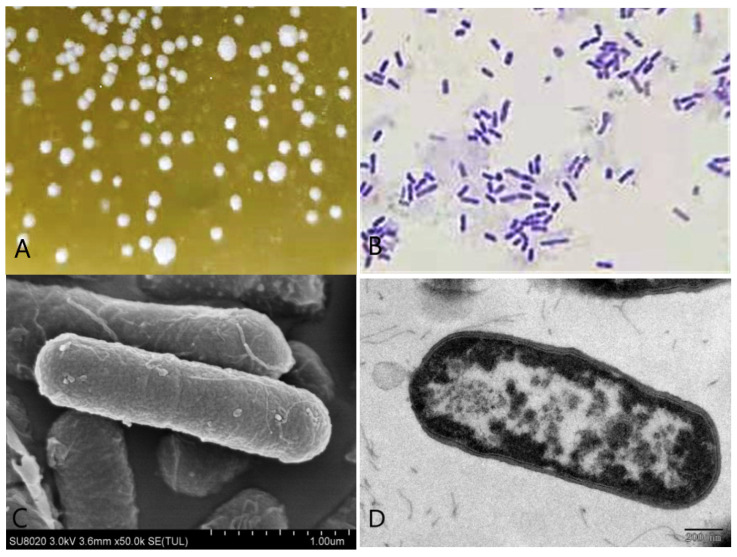
Morphological identification of the *L. agilis* WWK129 strain. (**A**) The colony characteristics of this strain; (**B**) the microstructure characteristics of this strain at 1000 magnification; (**C**) the scanning electron microscopical observation of this strain; (**D**) the transmission electron microscopical observation of this strain.

A single strain was isolated from the rumen liquid on basal medium plates containing 1 g/L gossypol as the sole carbon source after three days of anaerobic fermentation (Figure 3A), and 49.25% of gossypol in the medium disappeared. The appearance of colonies on the basal medium plates was rough and dirty white with wrinkly surfaces and irregular shapes. The bacteria were positive by Gram-staining (Figure 3B) and confirmed visible rod-shaped bacteria elements by both SEM and TEM observations (Figure 3C,D).

### 3.2. 16S rDNA Homology

**Figure 4:**
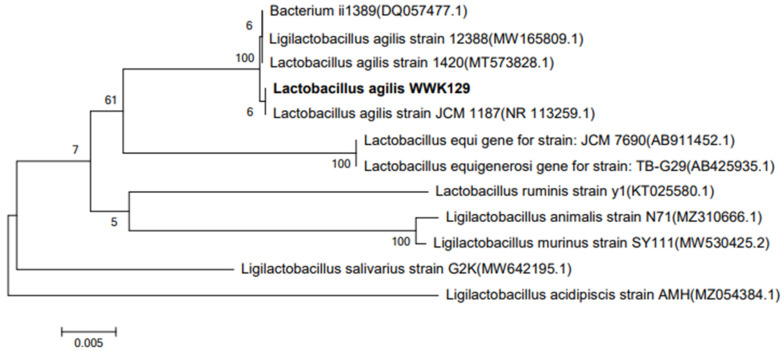
Phylogenetic tree based on 16S rDNA sequence of gossypol-degradation strain *L. agilis* WWK129.

The genomic DNA of the isolated strain was successfully extracted and amplified with a bacteria-specific primer pair, which generated an amplicon of about 1.5 kb and was used for the analysis of a range of bacterial strains. Sequence analysis showed the size of the amplicon was 1446 bp. The 16S rDNA sequences of isolated strain were aligned using the NCBI-BLAST database, indicating that it is 99.72% homologous to the sequence of *L. agilis* strain JCM 1187 (Figure 4). It was the first time that an *L. agilis* strain that could use gossypol as its sole carbon resource was isolated from the rumen, and it was named *L. agilis* WWK129 in the present study.

### 3.3. Growth Study

**Figure 5:**
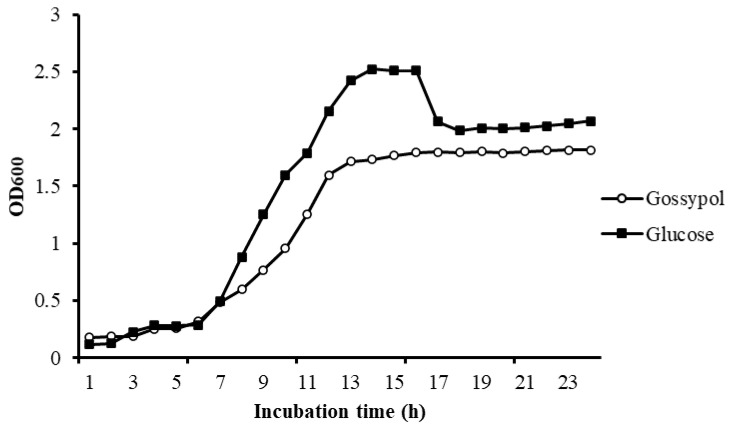
Growth curve of *L. agilis* WWK129 in carbon source of gossypol and glucose. OD600, optical density of incubated medium at 600 nm.

There is no obvious difference in bacteria density in different carbon sources in the lag phase. Bacteria in different mediums both exponentially increased after 6 h of incubation, but the maintenance time of the log phase in gossypol was significantly shorter than it in glucose. There was an obvious decline phase of bacteria in glucose but not in gossypol. The final bacteria density of *L. agilis* WWK129 in glucose was significantly greater than it in gossypol (Figure 5).

### 3.4. Gossypol Disappearance and Nutrient Shifts of Fermented CSM

**Figure 6:**
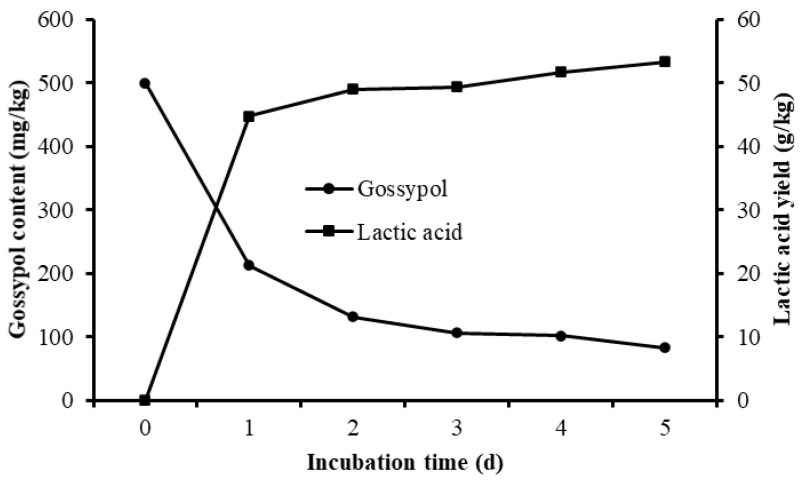
Gossypol content and lactic acid yield of cottonseed meal fermented by *L. agilis* WWK129.

Different culture periods were chosen to evaluate the effect of solid-state fermentation by *L. agilis* WWK129 strain on the nutritive value of CSM. Compared with the control, the gossypol content of fermented CSM decreased obviously, gossypol degradability increased significantly along with the prolongation of fermentation and it reached over 80% after five days of anaerobic fermentation (Figure 6). The NDF and ADF contents of CSM decreased significantly after fermentation (*p* < 0.01), while CP and lactic acid content were significantly higher than the control (*p* < 0.05) (Figure 6 and Table 1). It was evident that most amino acids of CSM improved markedly by fermentation of *L. agilis* WWK129, whereas Aspartic acid, Serine, Glutamic acid, Proline, and Arginine decreased. Among essential amino acids, levels of Phenylalanine increased the most, while among non-essential amino acids, Alanine increased the most.

## 4. Discussion

### 4.1. Identification of L. agilis WWK129

Many times, rumen microorganisms have been demonstrated to be capable of gossypol biodegradation ability. Chen et al. [28] and Zhang et al. [29] had isolated Bacillus strains from the rumen with high activity of gossypol degradation successively. In the present study, the strain named *L. agilis* WWK129 which could use gossypol as its sole carbon resource was isolated from the rumen of dairy cows and identified according to morphological and molecular methods. Our previous study had revealed that there was a promoting effect of gossypol on the activity of *Firmicutes* bacteria [30], and the isolated *Lactobacillus* belongs to *Firmicutes*, which was consistent with the result of previous research. Although *L. casei* and *L. plantarum* were found to degrade gossypol in previous studies [31,32], there was no report so far about gossypol biodegradation by *Lactobacillus* strain in the rumen, and this is the first time that a *Lactobacillus* strain could degrade gossypol isolated from the rumen in the present study.

*Lactobacillus* species are common constituents of gastrointestinal tracts [33], they have been used as direct-fed probiotics [34] and as silage inoculation [35]. The beneficial effects of *Lactobacillus* on the production performance of dairy cows included increase in milk yield, improvement of feed efficiency, and increase in daily weight gain [36]. In ruminants, *Lactobacillus* bacteria are generally only prevalent in young animals before the rumen has properly developed. To date, *Lactobacillus* bacteria commonly found in the rumen of adult dairy cows included *L. ruminis*, *L. vitulinus*, *L. acidophilus*, *L. casei*, *L. fermentum*, *L. plantarum*, *L. buchneri*, *L. brevis*, *L. cellobiosus*, *L. helveticus*, and *L. salivarius* [37]. *L. agilis* is firstly isolated from municipal sewage in 1981, and it is commonly known as motile *Lactobacillus* species from sewage and chicken guts [38,39], it was demonstrated that *L. agilis* played a key role in the degradation of sinigrin, cholesterol, and glucosinolates [40,41]. There was limited knowledge about its function in ruminants, Zhu et al. [42] had isolated several *L. agilis* from the feces of dairy calves, and found that these bacteria readily degraded long chain inulin. To date, this is the first time found that *L. agilis* presented the ability of gossypol degradation, the successful isolation of *L. agilis* from the rumen would be helpful for a better understanding of its detoxification mechanism of gossypol in the future.

The method of isolating bacteria from the rumen by screening medium is commonly used for research on the function of rumen bacteria. In the present study, *L. agilis* WWK129 statin was cultivated in a basal medium using gossypol as the only carbon source, and about 50% of gossypol in basal medium plates disappeared after three days of anaerobic fermentation, which demonstrated that gossypol could provide the carbon source for its growth by biodegradation rather than transformation. The exponential phase of growth curve is a stage in which microbial cells are divides at the maximal rate and the nature of the medium is being used as quickly as possible. According to the growth curve of *L. agilis* WWK129 in different carbon resources, the maintained time and slope of exponential phase in gossypol was smaller than glucose, which noted that *L. agilis* WWK129 grow faster in glucose instead of gossypol. It was assumed that a variety of environmental conditions cause the death of microorganisms, such as the depletion of essential nutrients in the decline phase. The optical density of the medium containing glucose decreased significantly after 16 h of incubation, this was mainly due to the decrease of nutrient composition of medium and the increase of cell metabolites which inhibited the growth of incubated bacteria. There was no decline phase in medium contained gossypol after 24 h of incubation and the final density of *L. agilis* WWK129 in stationary phase was greater in medium contained glucose, which revealed that the biodegradation and transformation rate of gossypol was relatively slow and difficult, and the utilization efficiency of gossypol was lower than glucose.

### 4.2. Effect of L. agilis WWK129 Fermentation on the Nutritional Value of CSM

In the present study, fiber content of control group inoculated with MRS medium without *L agilis* WWK129 were consistent with the original CSM sample, while CP content was significant higher than original state, this could be due to the peptone in MRS medium which improved the CP level of control group. According to the results of Chen et al. [28] and Zhang et al. [29], *Bacillus* strains isolated from the rumen applied in optimum fermentation conditions, the gossypol content in solid-state fermented CSM decreased by 80%, relative to the control. In the current study, it was found that over than 80% gossypol in CSM could be degraded by *L. agilis* WWK129 in solid-state fermentation after five days, which was relatively greater than previous research, it could be due to the different fermentation conditions, CSM variety, and gossypol biodegradation ability of stains. The disappearance of gossypol could be due to the biodegradation of microbial enzymes secreted by *L. agilis* WWK129 and gossypol, its metabolites could also be used by *L. agilis* WWK129 as a carbon resource as well. Wu [43] investigated the effect of solid-state fermentation by three types of *L. acidophilus* on the nutritional value of CSM and found that lactic acid and essential amino acids content of fermented CSM were greater than the control, which was consistent with the results of the present study. The improvement of CP and amino acids content in fermented CSM was mainly due to the growth of microflora, as well as the increase of cellular protein and microbial enzymes secreted by *L. agilis* WWK129, which could promote the nutritive value and utilization efficiency of protein in CSM. The fiber content of fermented CSM was significantly lower than that of CSM without inoculum, it was noted that *L. agilis* WWK129 could be helpful for the degradation of fiber, and the reduction of fiber could provide carbon resources for the growth of *L. agilis* WWK129. Lactic acid is a key end-product of *Lactobacillus* fermentation, and lactic acid content persistently increased along with the incubation reflected that the fermentation conditions in the present study were suitable for the growth of *L. agilis* WWK129.

Solid-state microbial fermentation is an important method used for gossypol detoxification in the cotton by-products process, and *Candida tropicalis* and *Lactobacillus plantarum* were commonly used in the fermentation of CSM feed sources in previous research. Zhang et al. [44] found that gossypol degradability in CSM fermented by *C. tropicalis* ZD-3 reached 94.6% after 48 h, CP content increased by about 11% compared with the control, total amino acids, and essential amino acids content of fermented CSM increased by 27% and 29%, respectively. Khalaf and Meleigy [15] investigated the effect of fungi on gossypol content of CSM by solid-state fermentation and found that *C. tropicalis* could degrade 86.18% gossypol after 72 h of incubation at 30 °C. Hong [45] found that gossypol degradability in CSM with 5% inoculum of *L. plantarum* reached 40% after 60 h solid-state fermentation at 30 °C. There is no report about feed resource fermented by *L. agilis* in previous, the results of the current study have verified the feasibility of *L. agilis* used in solid-state fermentation of CSM and provided more bacteria selection for the microbial fermentation of cotton by-products in the future.

*Lactobacillus* strains were approved for use as probiotics for both humans and animals [46,47], and the colonization of *Lactobacillus* strains in the gut epithelium could reduce the risk of pathogenic bacterial infection by the production of lactic acid and other metabolites [48]. Lactic acid in fermented CSM feed sources can increase acidity, inhibit the growth of spoilage bacteria and prevent the mildew of feed. In addition, all of this research demonstrated that the fermentation of *Lactobacillus* strain can significantly increase the content of essential amino acids and improve aminol acids balance. Consequently, the use of *L. agilis* WWK129 in CSM fermentation can not only reduce the toxicity of gossypol and promote nutritive value, but also be conducive to the health of animals.

## 5. Conclusions

Collectively, the present study noted that *L. agilis* WWK129, firstly isolated from the rumen contents, presented the great potential for gossypol biodegradation. Gossypol disappearance rate in cottonseed meal reached up to 80% after 5 days of anaerobic fermentation by the isolated strain, meanwhile, neutral detergent fiber and acid detergent fiber content decreased by about 4% and 5%, respectively, crude protein content increased by about 4% and most essential amino acids content improved significantly, suggesting that the *L. agilis* WWK129 has promising application prospect in gossypol detoxification and microbial fermentation of cottonseed meal, and it could provide a basis for further analysis of gossypol biodegradation in the rumen.

## Figures and Tables

**Table 1:** Nutritive value of cottonseed meal fermented by *L. agilis* WWK129 (%, DM).

Item ^1^	Fermented CSM at Different Incubation Time						S.E.M	*p* Value		
	Con ^2^	1 Day	2 Day	3 Day	4 Day	5 Day		Time	L	Q
NDF	16.3 ^a^	15.3 ^ab^	14.9 ^ab^	14.6 ^ab^	13.6 ^bc^	12.7 ^c^	0.46	<0.01	0.45	0.04
ADF	11.3 ^a^	6.8 ^b^	6.5 ^c^	6.4 ^cd^	6.3 ^de^	6.1 ^e^	0.05	<0.01	<0.01	<0.01
CP	57.6 ^b^	60.2 ^a^	60.7 ^a^	60.9 ^a^	61.3 ^a^	61.7 ^a^	0.41	0.03	0.81	<0.01
Aspartic acid	4.28 ^a^	4.24 ^ab^	4.24 ^ab^	4.20 ^bc^	4.17 ^d^	4.17 ^d^	0.011	<0.01	0.29	<0.01
Threonine	1.29 ^c^	1.36 ^c^	1.36 ^c^	1.44 ^b^	1.51 ^a^	1.55 ^a^	0.020	<0.01	<0.01	0.01
Serine	1.77 ^a^	1.64 ^b^	1.65 ^b^	1.65 ^b^	1.65 ^b^	1.67 ^b^	0.028	0.10	0.42	0.20
Glutamic acid	11.36 ^a^	10.74 ^ab^	10.52 ^b^	10.42 ^b^	10.32 ^b^	9.94 ^b^	0.222	0.04	0.50	0.01
Proline	1.46 ^a^	1.28 ^b^	1.27 ^b^	1.19 ^b^	1.07 ^c^	0.99 ^c^	0.029	<0.01	0.11	<0.01
Glycine	1.87	1.90	1.91	1.92	1.93	1.99	0.034	0.36	0.31	0.13
Alanine	1.76 ^c^	1.78 ^c^	1.80 ^c^	1.87 ^b^	1.98 ^a^	1.98 ^a^	0.021	<0.01	<0.01	0.10
Cysteine	0.74 ^b^	0.74 ^b^	0.76 ^ab^	0.78 ^ab^	0.79 ^a^	0.79 ^a^	0.016	0.09	0.07	0.09
Valine	2.11 ^d^	2.18 ^cd^	2.35 ^bc^	2.40 ^ab^	2.48 ^ab^	2.54 ^a^	0.050	<0.01	0.01	<0.01
Methionine	0.56 ^c^	0.60 ^c^	0.61 ^c^	0.68 ^b^	0.74 ^a^	0.75 ^a^	0.016	<0.01	<0.01	0.01
Isoleucine	1.53 ^d^	1.53 ^d^	1.58 ^cd^	1.64 ^bc^	1.67 ^ab^	1.73 ^a^	0.019	<0.01	0.01	<0.01
Leucine	2.71 ^d^	2.79 ^c^	2.80 ^c^	2.85 ^b^	2.87 ^ab^	2.91 ^a^	0.033	<0.01	<0.01	<0.01
Tyrosine	1.52 ^c^	1.53 ^bc^	1.56 ^abc^	1.58 ^ab^	1.60 ^a^	1.61 ^a^	0.015	0.02	0.06	0.05
Phenylalanine	2.53 ^d^	2.75 ^c^	2.81 ^bc^	2.90 ^b^	3.21 ^a^	3.21 ^a^	0.037	<0.01	<0.01	<0.01
Lysine	1.90 ^b^	1.91 ^b^	1.93 ^b^	2.18 ^a^	2.28 ^a^	2.31 ^a^	0.044	<0.01	<0.01	0.04
Histidine	1.27	1.25	1.27	1.28	1.27	1.27	0.016	0.59	0.19	0.35
Arginine	5.66 ^a^	5.53 ^ab^	5.44 ^ab^	5.47 ^ab^	5.21 ^bc^	5.09 ^c^	0.089	0.03	0.40	0.12

^a,b,c,d,e^ Values in a line within the same class without a common superscript are significantly different (*p* < 0.05). ^1^ ADF, acid detergent fiber; CP, crude protein; NDF, neutral detergent fiber; L, linear; Q, quadratic. ^2^ Con, cottonseed meal inoculated with MRS medium without the isolated strain to prevent the confusion of the effect of medium contents on fermentation results.

## Data Availability

Data from the study are available in NCBI-GenBank under accession number MZ 779227.
